# Practical Notes on Diagnosis and Treatment

**Published:** 1907-01-26

**Authors:** 


### Practical Notes on Diagnosis and Treatment.

**Dilatation of Sphincter Ani.**

Many cases of obstinate constipation, especially in women, are due to tightness of the sphincter ani, or, to speak more carefully, are relieved by forcible manual dilatation of it.—*Professor Clifford Allbutt.*

**Iron in Chlorosis.**

Niemeyer's statement that three boxes of ninety-six Blaud's pills will cure any case of chlorisis is a very misleading one. The great principle of treatment is not merely to treat actively, but by long continuance. In order to secure a cure the treatment must be as protracted as the disease.—*Sir Stephen Mackenzie.*

**An Ice Pack.**

The patient, having been stripped, is placed on a mackintosh sheet, and an ordinary cotton sheet, folded so as to have four layers, is wrung out in iced water, and the interspaces between the layers are packed with powdered ice. This pack is wrapped round the body with the limbs left free, and in such a way that the axillæ are accessible to thermometrical observation.

**Inoperable Uterine Cancer.**

Dr. Bernhart has used injections of salicylic acid in cases of cancer of the cervix beyond the reach of surgical interference. He uses a 6 per cent. solution of the acid in a 60 per cent. alcoholic menstruum. Of this about half a drachm is employed at a sitting, a few drops being injected into the growth at six or seven points. This application is made every four or five days.

**Acne.**

When there is much pustulation one of the most useful applications is one which Erasmus Wilson employed largely—a dram of the hypochlorite of sulphur to an ounce of lard. This is to be rubbed into the pustules at night. Care must, however, be taken to see that it is made with the powdered hypochlorite and that it is fresh.—*Dr. Radcliffe Crocker.*

**Carbohydrates in Diabetes.**

Every diabetic should be allowed as much carbohydrate as he is able to assimilate, estimating this by the amount of urine and sugar, and by the body weight. Milk (not exceeding a pint a day) and potatoes (to the amount of two or three large ones daily) may often be added to the diet with very little, if any, increase in the polyuria and glycosuria, and with benefit to the patient.—*Dr. Robt. Saundby.*

**Facial Paralysis.**

In recent cases hot fomentations applied over the ear and its neighbourhood constitute a very useful measure. They should be used for one-quarter of every hour during the first day. A blister should be applied over the mastoid process, and should be repeated as soon as the condition of the skin permits; but never put a blister in front of the ear over the place of exit of the nerve.—*Sir Wm. Gowers.*

**Diet in Cardiac Disease.**

One of the greatest services rendered by the late Sir Andrew Clark was his enforcement of the doctrine of spareness of diet in people suffering from heart disease.—*Dr. Sydney Coupland.*

**Mitral Stenosis.**

Sir Jas. Barr advocates the combined use of atropine and nitro-glycerine in this form of valvular disease. Half a minim, each, of solution of sulphate of atropine and of solution of trinitrin may be given thrice daily.

**Erysipelas of the Face.**

In this condition Dr. Tison advises painting of the affected part with a saturated ethereal solution of camphor. This acts as a sedative and rapidly relieves the pain and inflammation. The application may be repeated as often as necessary. Obviously its inflammable nature calls for precautions.

**Peripheral Neuritis.**

In the treatment of this condition, and in addition to massage and electricity, strychnine given hypodermically is most useful. This may be commenced with 1 minim of a 1 per cent. solution of the nitrate, the dose being increased by 1 minim each day until a maximum of 10 minims is reached. Then there should be a few days' intermission, after which the maximum dose may be repeated for a week, then to be followed by a second intermission.

**Arsenic in Chorea.**

The medical treatment of chorea is arsenic in full doses. By full doses I mean 15 minim doses of Fowler's solution given three times a day for three days consecutively. The administration is then suspended for one or two days to see what the effect is generally. By suspending the remedy and then resuming it for a few days more, the effect produced is more marked. The more violent forms of choreic spasm are best met by chloral hydrate in full doses. *Sir Dyce Duckworth.*

**Ringworm of the Body and Beard.**

A suitable ointment is sublimed sulphur, 1 dram; ammoniated mercury, ½ dram; lanoline, 1 ounce. This is rubbed into the part twice a day. With this method of treatment you can state positively that in ringworm of the body you can cure it in a short time; and in ringworm of the beard you can cure it, but not in so short a time, because the difficulty is for the parasiticide to act closely and intimately on the part affected.—*Dr. Allan Jamieson.*

**Alcohol in Enteric Fever.**

The chief indications on which I prescribe alcohol are: (1) a weak first sound of the heart, (2) dicrotic pulse, (3) wandering delirium, (4) involuntary passage of fæces and urine. The late Dr. Murchison taught that eight ounces of brandy daily was always enough, but there are some cases in which much more must be given, and in which I feel sure a much freer use has saved the patient's life.—*Dr. Curnow.*

